# Extreme Mechanical Properties of Regular Tensegrity Unit Cells in 3D Lattice Metamaterials

**DOI:** 10.3390/ma13214845

**Published:** 2020-10-29

**Authors:** Anna Al Sabouni-Zawadzka

**Affiliations:** Faculty of Civil Engineering, Warsaw University of Technology, Al. Armii Ludowej 16, 00-637 Warsaw, Poland; a.sabouni@il.pw.edu.pl

**Keywords:** tensegrity, lattice metamaterial, elasticity, extreme properties

## Abstract

The study focuses on the identification of extreme mechanical properties of 3D lattice metamaterials based on regular tensegrity modules: 4-strut simplex, 3-strut simplex, expanded octahedron, truncated tetrahedron and X-module. The basis of the analysis is a continuum model which is used to find the equivalent elasticity matrices of the unit cells. For each examined tensegrity module a line of extreme properties is determined, which indicates the occurrence of the soft mode of deformation. Moreover, the eigenvectors corresponding to soft and stiff deformation modes are calculated and presented graphically. The obtained results are promising from the point of view of future creation of tensegrity lattices and metamaterials with extreme mechanical properties. One of the analysed materials is identified as quasi bimode, two as quasi trimodes, another one as a trimode and one more as a unimode.

## 1. Introduction

The idea of extreme materials was developed in 1995 by Milton and Cherkayev [1]. The term extreme implies that the material is extremely stiff under certain stresses or extremely compliant in other orthogonal cases of stresses. Some of extreme materials exhibit unusual mechanical properties, such as for example a negative Poisson’s ratio. Extreme material properties may be examined by analysing the elasticity tensor, which can be diagonalized using the orthogonal transformation. After diagonalization, a set of eigenvalues is obtained, with orthogonal eigenvectors that describe deformation forms of the analysed material. The number of eigenvalues that are close to zero indicates the type of extreme material, which can be: nullmode, unimode, bimode, trimode, quadramode, pentamode or hexamode [1,2].

Analysis of extreme mechanical properties of tensegrity systems remains a compelling challenge in the field of mechanics of space latices or engineering metamaterials [3,4]. The concept of tensegrity metamaterials was introduced in [5], where the dynamic properties of the set of tensegrity prisms are analysed, and then developed in [6,7,8]. Self-similar tensegrity masts of order 1 and higher are discussed in [9]. The work [10] focuses on the morphological optimization of tensegrity-like metamaterials with a novel model of an infinite slab. In [11], the authors describe extreme softening/stiffening response of axially loaded tensegrity prisms exhibiting the geometrically nonlinear behaviour. They focus on the design and manufacture of tensegrity lattices and innovative metamaterials. The papers [12,13] propose three-dimensional tensegrity lattices constructed from basic tensegrity octahedron modules used as unit cells. Another interesting approach is presented in [14,15], where tensegrity cell mechanical metamaterial with metal rubber is proposed. In [16] the authors develop an orthotropic metamaterial built from tensegrity unit cells. They prove that the proposed mechanical metamaterial has a negative Poisson’s ratio and some properties which can be regarded as smart [17,18,19]. To summarize, it can be noticed from the literature study that there has been a significant development of tensegrity latices and metamaterials in recent years.

Identification of mechanical properties of tensegrity metamaterials can be performed using a continuum model [20,21]. It is based on the equivalence of the strain energy defined within the 3D theory of elasticity and the strain energy in a discrete formulation. Such an approach includes nonlinearities corresponding to the equations of equilibrium in actual configuration of the structure with self-stress. The paper [21] focuses on the evaluation of equivalent mechanical properties of various tensegrity modules using the continuum model. It is explained there how to build the continuum model for tensegrities and how to determine technical coefficients with the presented approach. Using the adopted model, mechanical characteristics are determined and graphs of identified mechanical coefficients for five typical tensegrity modules are presented.

A similar approach is used in the paper [22], which is dedicated to the evaluation of extreme properties of cellular metamaterials based on the simplex tensegrity pattern. Supercells constructed from simplex modules with various geometrical proportions are analyzed. The concept of extreme properties is described in short and the continuum model described in [20,21] is applied to identify mechanical properties of the considered structures. The eigensolution of the equivalent elasticity matrices of the continuum model is used to define six representative deformation modes.

In the analyses presented in this manuscript the continuum model, which was proposed in [20,21] and used in [22], is applied as a method of analysis that allows us to determine technical coefficients and estimate elastic properties of the structures. However, the continuum model itself is used here only as a tool and is described in short in Section 2. The novelty of this study is a methodical search of material structures with extreme mechanical features. Five regular tensegrity modules are considered: 4-strut simplex, 3-strut simplex, octahedron, tetrahedron and X-module. It is proved that the analyzed systems can be regarded as extreme and the parameters for which it occurs are given. It is not a trivial task, and in fact very few structures have extreme properties—for example in trusses without infinitesimal modes no extreme features can be identified. The original results presented in this paper, which include determination of the parameters that assure occurrence of extreme properties and identification of stiff and soft modes, are based on the previously published results [21] on the continuum description of tensegrity modules. The author decided to repeat some of the formulas presented in [21] to make this work clear and enable the readers to repeat or verify the original results of this study.

The applied continuum model can be used for the analysis of both tensegrity unit cells and lattice structures or materials composed of these cells. As it is proved in the next section of this paper, under certain assumptions (compliance of infinitesimal mechanisms) the properties of the cellular metamaterial are the same as the properties of its single cell. Therefore, in order to identify extreme mechanical properties of 3D lattice metamaterials, the author examines single tensegrity modules that can be applied as unit cells in mechanical metamaterials. As the results obtained for single modules correspond to the properties of materials constructed from them, the conclusions can be drawn in regard to whole lattice metamaterials.

## 2. Equivalent Mechanical Properties of Tensegrity Structures

The continuum model proposed in [20] and analysed in [21] is based on the equivalence of the strain energy of an unsupported tensegrity structure defined with the use of the finite element method (FEM) [23,24,25,26] and the strain energy of a solid determined using the symmetric linear 3D elasticity theory (LTE) [27]. The strain energy of a tensegrity truss according to FEM is dependent on the global linear stiffness matrix $K_{L}$ and the global geometric stiffness matrix $K_{G}=S_{0}K_{g}$. The self-equilibrated set of normal forces of the structure with the multiplier $S_{0}$ is represented by the second of the above matrices. The well known finite element formulation is based on a local approximation of the displacement field of a separate finite element with the use of shape functions. For the two-node truss finite elements the shape functions are formally exact and no approximation error is generated. The global stiffness matrix and geometric stiffness matrix are obtained with the use of local matrices after the standard process of globalization [23,26]. The global matrices required for the analysis can be also obtained in an algebraic way [28,29,30,31]. The algebraic formulation is global from the beginning of the analysis with no approximation and is based on creation of a compatibility matrix as a relation between extensions of truss members and global displacements of the structure. Elastic and geometric data of the structure are represented with a separate diagonal matrix. The two formulations lead to the same results (linear stiffness matrix [28,29,30] and geometric stiffness matrix [31]), however the FEM formalism is recommended for the 3D models [30] as it is simpler to algorithmize and program.

The technique used in the continuum model (for details see [20,21] and papers cited therein) leads to the equivalent symmetric elasticity matrix $E=[E_{i,j}],i,j=1,...,6$, according to the LTE theory. Description and detailed discussion of the equivalent mechanical properties of tensegrity modules based on the continuum model is published in [21]. The applied model is non-linear in the sense of equilibrium equations considered in actual configuration. Validation of the model for structures with self-stress is presented in the annex to [22].

The applied continuum model can be used for the analysis of simple unit cells as well as more complicated lattice structures or materials composed of such cells. It is proved below that depending on the arrangement of single cells in the material, the properties of the material remain the same or become different from the features of the single module.

**Figure 1:**
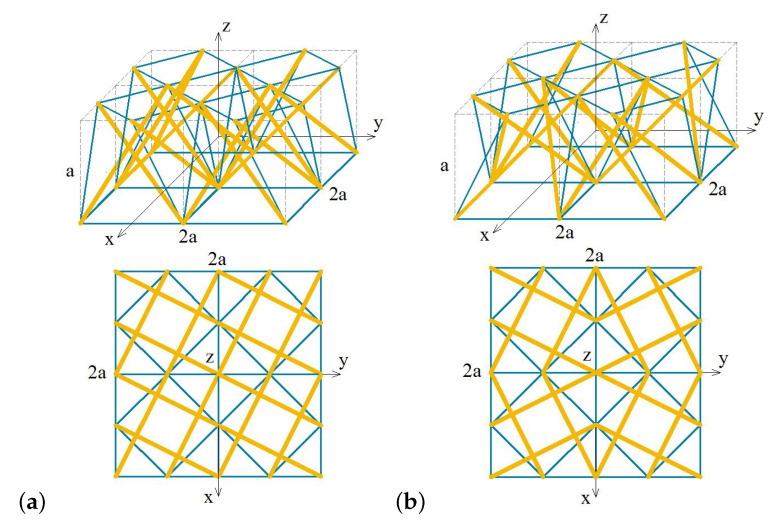
Two configurations of a 4-unit supercell: (**a**) A—anisotropic configuration. (**b**) B—orthotropic configuration. Struts are marked with thick yellow lines and cables with thin blue ones.

In the case of a 4-strut simplex, the modules can be connected in accordance with their infinitesimal mechanisms or not. In Figure 1 two configurations of a 4-module supercell based on the 4-strut simplex module are presented:

- Configuration A (Figure 1a)—an anisotropic layout with four modules rotated clockwise;
- Configuration B (Figure 1b)—an orthotropic layout with two modules rotated clockwise and two counterclockwise to obtain a symmetry.

The presented supercells consist of four tensegrity modules connected in nodes of their upper and lower bases. The single unit cell is a 4-strut simplex inscribed into a cube of edge length *a* and therefore, the volume of the supercell in the formula for the strain energy of a solid determined according to LTE (see [22] for details) should be taken as $4a^{3}$.

The elastic matrix E obtained from the continuum model for the anisotropic configuration A has a form

(1) $$\begin{array}{cc} \; & E_{A}=\left[\begin{array}{cccccc} e_{11} & e_{12} & e_{13} & e_{14} & 0 & 0\\ \; & e_{11} & e_{13} & -e_{14} & 0 & 0\\ \; & \; & e_{33} & 0 & 0 & 0\\ \; & \; & & e_{12} & 0 & 0\\ \; & \; & & \; & e_{13} & 0\\ \mathrm{sym}. & \; & & \; & & e_{13} \end{array}\right],\\ & e_{11}=\frac{2EA}{a^{2}}\left(0.314815+1.39827\cdot k-0.0794978\cdot \sigma \right),\\ \; & e_{12}=\frac{EA}{a^{2}}\left(0.296296+0.707107\cdot k-0.0134742\cdot \sigma \right),\\ \; & e_{13}=\frac{EA}{a^{2}}\left(0.740741+0.357771\cdot k+0.17247\cdot \sigma \right),\\ \; & e_{14}=\frac{EA}{a^{2}}\left(-0.222222-0.0808452\cdot \sigma \right),\\ \; & e_{33}=\frac{2EA}{a^{2}}\left(0.592593+1.43108\cdot k-0.17247\cdot \sigma \right), \end{array}$$

where:

$k={\left(EA\right)}_{\mathrm{cable}}/{\left(EA\right)}_{\mathrm{strut}},{\left(EA\right)}_{\mathrm{strut}}=EA,\sigma =S_{0}/EA$,

$S_{0}$—multiplier of self-stress forces,

*E*—elastic modulus of the strut material,

*A*—cross-section of the strut.

The same matrix determined for the orthotropic configuration B takes a form

(2) $$\begin{array}{cc} \; & E_{B}=\left[\begin{array}{cccccc} e_{11} & e_{12} & e_{13} & 0 & 0 & 0\\ \; & e_{11} & e_{13} & 0 & 0 & 0\\ \; & \; & e_{33} & 0 & 0 & 0\\ \; & \; & & e_{12} & 0 & 0\\ \; & \; & & \; & e_{13} & 0\\ \mathrm{sym}. & \; & & \; & & e_{13} \end{array}\right],\\ & e_{11}=\frac{2EA}{a^{2}}\left(0.314815+1.13709\cdot k-0.0794978\cdot \sigma \right),\\ \; & e_{12}=\frac{EA}{a^{2}}\left(0.296296+0.707107\cdot k-0.0134742\cdot \sigma \right),\\ \; & e_{13}=\frac{EA}{a^{2}}\left(0.740741+0.268328\cdot k+0.17247\cdot \sigma \right),\\ \; & e_{33}=\frac{2EA}{a^{2}}\left(0.592593+1.07331\cdot k-0.17247\cdot \sigma \right). \end{array}$$

It can be noticed that matrix $E_{A}$ (Equation (1)) is identical to the elastic matrix obtained for the 4-strut simplex module (see Section 4). Matrix $E_{B}$ (Equation (2)), on the other hand, differs from the the elastic matrix of the unit cell. It can therefore be concluded that depending on the arrangement of single cells in the material, the properties of the material remain the same or become different from the features of the single module.

**Figure 2:**
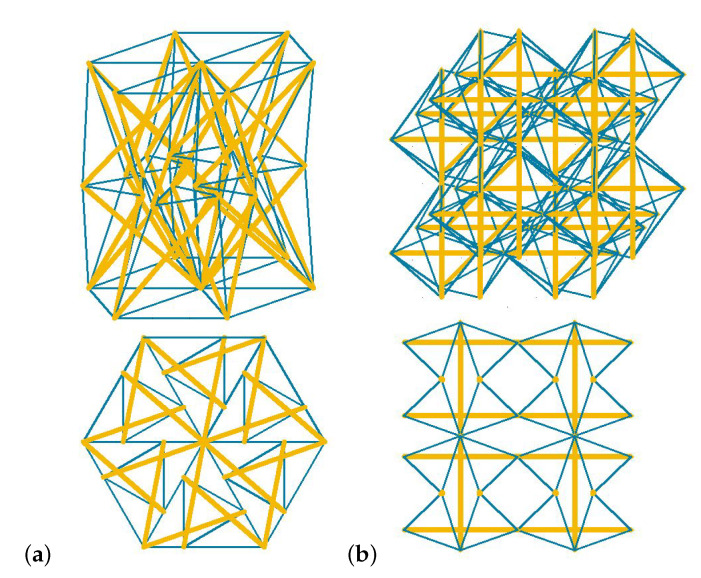
Material patterns: (**a**) 3-strut simplex pattern. (**b**) Expanded octahedron pattern. Struts are marked with thick yellow lines and cables with thin blue ones.

In the present paper it is assumed that the materials are constructed similarly to the configuration A of the supercell—all modules should be arranged in accordance with their infinitesimal mechanisms to form a structure that has identical properties as its unit cells analysed separately. The formation of a material with a kinematic compatibility between the infinitesimal mechanisms of its unit cells is a complicated problem that has already been addressed by Motro [32]. Examples of such configurations of the 3-strut simplex and expanded octahedron patterns are shown in Figure 2. In the truncated tetrahedron and X modules the task becomes even more complicated and can be a subject of further investigation. It may lead to both modular systems and new tensegrities, for example with additional cables.

## 3. Extreme Material

The idea of extreme materials was introduced in [1]. Extreme means: extremely stiff under certain stresses or extremely compliant in other orthogonal cases of stresses. Some of extreme materials exhibit unusual mechanical properties, such as a negative Poisson’s ratio. Extreme material features can be identified by examining the elasticity tensor, which is positive definite and shows certain symmetries. The mentioned tensor can be diagonalized using the orthogonal transformation. The diagonal representation of the tensor noted in the Voight’s form—as a square 6 × 6 matrix E—is a set of eigenvalues $\lambda_{i}>0$(i=1,2,...,6). The corresponding orthogonal eigenvectors $w_{i}$ describe deformation forms of the analysed material. The number of eigenvalues that are close to zero indicates the type of extreme material, which can be classified as: nullmode, unimode, bimode, trimode, quadramode, pentamode or hexamode [1,2]. In the analyses presented in this study it is assumed, that the eigenvalue is close to zero if it is smaller than the maximum eigenvalue by at least four orders of magnitude. Traditional materials are usually nullmode. The proposed classification can be used for the determination of material properties as long as the elastic matrix E is known.

**Figure 3:**
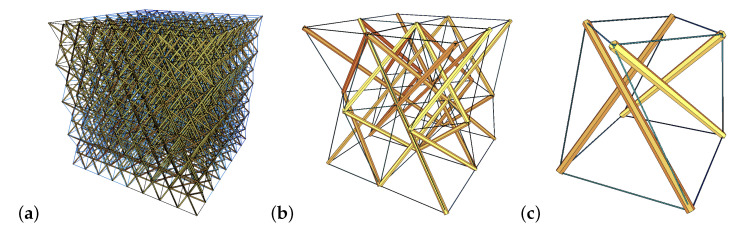
3D lattice metamaterial based on the tensegrity pattern: (**a**) Micro-scale—material. (**b**) Medium-scale—an 8-module supercell. (**c**) Macro-scale—a unit cell. Struts are marked with yellow color and cables with thin blue lines.

In the present paper the authors use the continuum model [20,21,22], to determine the elastic matrix E and thus, to examine the possibility of occurrence of extreme properties. As it was proved in the previous section, properties of the cellular metamaterials considered in this paper are the same as the properties of their single cells. Therefore, in order to identify extreme mechanical properties of proposed 3D lattice metamaterials, the authors examine single tensegrity modules. In individual tensegrity modules there are two parameters *k* and σ (described in detail in the previous section), which can be used to control the properties of the matrix E while searching for soft- and stiff modes. If a material is constructed in such a way that there maintains a compliance of infinitesimal mechanisms on the micro- (Figure 3a) or medium-scale (Figure 3b), then the features of the equivalent matrix E remain the same as for the single module (Figure 3c).

In the next section a methodical search of materials with extreme mechanical properties is presented. Five regular tensegrity modules are considered: 4-strut simplex, 3-strut simplex, octahedron, tetrahedron and X-module. The proposed tool is general and can be used to analyse the features of materials and structures with any arrangement of modules. However, this requires constructing a continuum model for each analysed system. Examples of such analyses can be found in [22].

## 4. Extreme Properties of Regular Tensegrity Modules

In this section, a study of extreme properties of five regular tensegrity modules inscribed into a cube of edge length *a* is presented. The analyses are based on the control of two parameters *k* and σ (defined in Section 2) that can be adjusted in search of extreme properties of metamaterials.

The following features are described for each module:

- Equivalent elasticity matrix;
- Extreme properties:
  - The line on the plane k,σ on which the smallest eigenvalue of the elasticity matrix is close to zero—the line indicates the possible occurrence of the soft mode of deformation (the arrow shows the half-plane for which the matrix E is positive definite);
  - An example of the distribution of eigenvalues for k=0.1, scaled so that the volumes of all modules are identical and equal to the volume of the 4-strut simplex module, assuming the same material of cables and struts—the applied scaling allowed the authors to compare the stiff modes between the modules;
  - Eigenvectors corresponding to individual eigenvalues of the matrix E—the eigenvectors corresponding to soft and stiff deformation modes are presented in the drawings.

In the drawings presented in this section, struts are marked with thicker lines and cables with thinner ones.

### 4.1. 4-Strut Simplex

**Figure 4:**
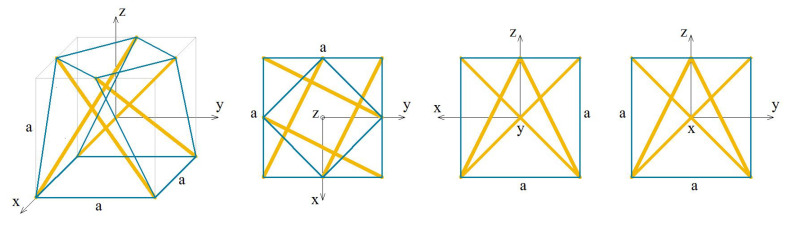
Geometry of the 4-strut simplex module.

The analysed 4-strut simplex module (S4) is presented in Figure 4.

The elastic matrix obtained from the continuum model has a form:

(3) $$\begin{array}{cc} \; & E_{S4}=\left[\begin{array}{cccccc} e_{11} & e_{12} & e_{13} & e_{14} & 0 & 0\\ \; & e_{11} & e_{13} & -e_{14} & 0 & 0\\ \; & \; & e_{33} & 0 & 0 & 0\\ \; & \; & & e_{12} & 0 & 0\\ \; & \; & & \; & e_{13} & 0\\ \mathrm{sym}. & \; & & \; & & e_{13} \end{array}\right],\\ & e_{11}=\frac{2EA}{a^{2}}\left(0.314815+1.39827\cdot k-0.0794978\cdot \sigma \right),\\ \; & e_{12}=\frac{EA}{a^{2}}\left(0.296296+0.707107\cdot k-0.0134742\cdot \sigma \right),\\ \; & e_{13}=\frac{EA}{a^{2}}\left(0.740741+0.357771\cdot k+0.17247\cdot \sigma \right),\\ \; & e_{14}=\frac{EA}{a^{2}}\left(-0.222222-0.0808452\cdot \sigma \right),\\ \; & e_{33}=\frac{2EA}{a^{2}}\left(0.592593+1.43108\cdot k-0.17247\cdot \sigma \right). \end{array}$$

**Figure 5:**
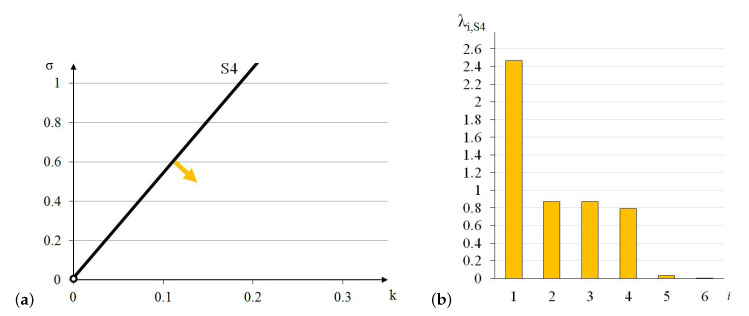
Extreme properties of the 4-strut simplex: (**a**) Line of extreme properties σ=0.012+5.34·k. (**b**) Distribution of eigenvalues for k=0.1,σ=0.546 (multiplier $EA/a^{2}$).

The elastic matrix indicates that the 4-strut simplex is an anisotropic module. Extreme properties of the module are presented in Figure 5.

The module can be identified as quasi bimode since one eigenvalue is close to zero and the second is much smaller than the other four. One positive eigenvalue is dominant over the others.

Analysis of the graphs (Figure 5) leads to the conclusion that the material based on the 4-strut simplex modules, arranged in accordance with their infinitesimal mechanisms, would have one stiff, two soft (one purely soft with the corresponding eigenvalue less than 0.01% of $\lambda_{\max,S4}$ and one quasi-soft with the eigenvalue less than 1.3% of $\lambda_{\max,S4}$) and three medium (eigenvalues around 32% and 35% of $\lambda_{\max,S4}$) modes of deformation. Moreover, realization of such a material is possible with the currently available materials.

**Figure 6:**
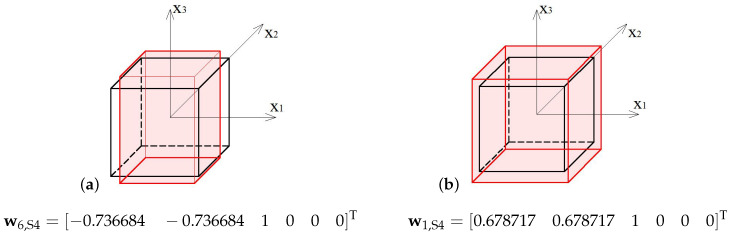
Deformation modes of the 4-strut simplex: (**a**) Soft mode. (**b**) Stiff mode.

The soft mode (Figure 6a) of the 4-strut simplex (S4), represented by the eigenvector $w_{6,S4}$, is volumetric with various signs, and the extension in $x_{3}$ direction exceeds by 35% the contraction in other directions. The stiff mode (Figure 6b), represented by the eigenvector $w_{1,S4}$, is volumetric with the uniform sign, and the extension in $x_{3}$ direction is 47% bigger than in others.

### 4.2. 3-Strut Simplex

**Figure 7:**
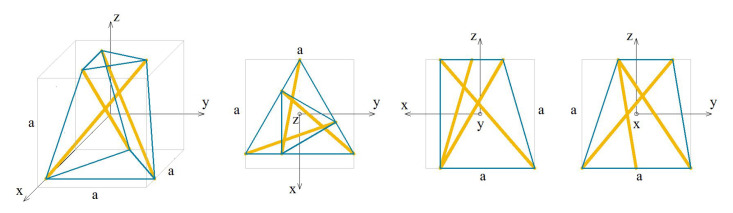
Geometry of the 3-strut simplex module.

The analysed 3-strut simplex module (S3) is presented in Figure 7.

The elastic matrix obtained from the continuum model has a form:

(4) $$\begin{array}{cc} \; & E_{S3}=\left[\begin{array}{cccccc} 3e_{12} & e_{12} & e_{13} & 0 & e_{15} & e_{16}\\ \; & 3e_{12} & e_{13} & 0 & -e_{15} & -e_{16}\\ \; & \; & e_{33} & 0 & 0 & 0\\ \; & \; & & e_{12} & e_{16} & -e_{15}\\ \; & \; & & \; & e_{13} & 0\\ \mathrm{sym}. & \; & & \; & & e_{13} \end{array}\right],\\ & e_{12}=\frac{EA}{a^{2}}\left(0.0957031+0.595459\cdot k-0.0400226\cdot \sigma \right),\\ \; & e_{13}=\frac{EA}{a^{2}}\left(0.492188+0.142302\cdot k+0.16009\cdot \sigma \right),\\ \; & e_{15}=\frac{EA}{a^{2}}\left(0.182677+0.0770235\cdot \sigma \right),\\ \; & e_{16}=\frac{EA}{a^{2}}\left(-0.117187-0.0237171\cdot k-0.0415049\cdot \sigma \right),\\ \; & e_{33}=\frac{2EA}{a^{2}}\left(0.632813+1.28072\cdot k-0.16009\cdot \sigma \right). \end{array}$$

**Figure 8:**
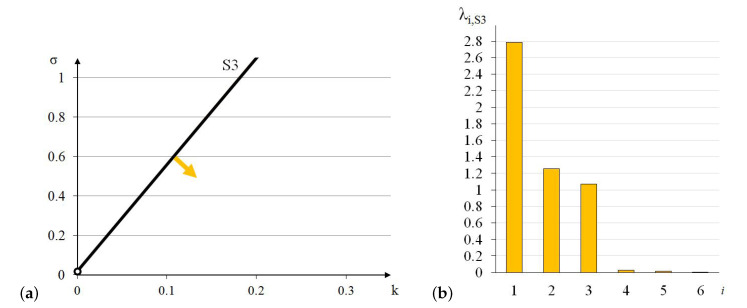
Extreme properties of the 3-strut simplex: (**a**) Line of extreme properties σ=0.023+5.4·k. (**b**) Distribution of eigenvalues for k=0.1,σ=0.563 (multiplier $EA/a^{2}$).

Similarly to the previous module, the elastic matrix indicates that the 3-strut simplex is anisotropic. Extreme properties of the module are presented in Figure 8.

The module can be identified as quasi trimode since one eigenvalue is close to zero and two others are much smaller than the other three. One positive eigenvalue is dominant over the others.

Analysis of the graphs (Figure 8) leads to the conclusion that the material based on the 3-strut simplex modules, arranged in accordance with their infinitesimal mechanisms, would have one stiff, three soft (one purely soft with the corresponding eigenvalue less than 0.01% of $\lambda_{\max,S3}$ and two quasi-soft with eigenvalues less than 1% of $\lambda_{\max,S3}$) and two medium (eigenvalues around 38% and 45% of $\lambda_{\max,S3}$) modes of deformation. Moreover, realization of such a material is possible with the currently available materials.

**Figure 9:**
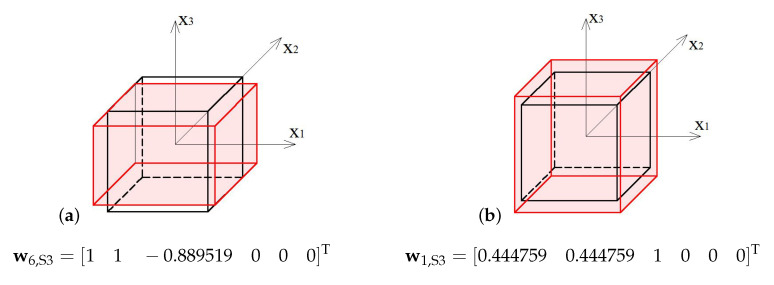
Deformation modes of the 3-strut simplex: (**a**) Soft mode. (**b**) Stiff mode.

The soft mode (Figure 9a) of the 3-strut simplex (S3), represented by the eigenvector $w_{6,S3}$, is volumetric with various signs, and the contraction in $x_{3}$ direction is 11% lower than the extension in other directions. The stiff mode (Figure 9b), represented by the eigenvector $w_{1,S3}$, is volumetric with the uniform sign, and the extension in $x_{3}$ direction is 125% bigger than in others.

### 4.3. Expanded Octahedron

**Figure 10:**
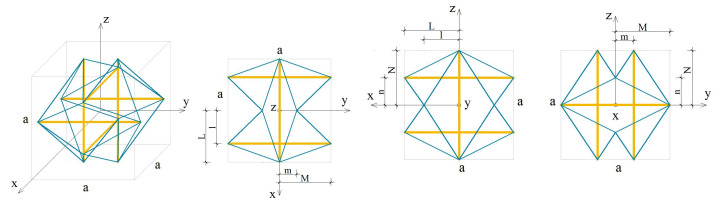
Geometry of the expanded octahedron module, with adopted parameters: l/L=0.65,m/M=0.30,n/N=0.56.

The analysed expanded octahedron module (O) is presented in Figure 10.

The elastic matrix obtained from the continuum model has a form:

(5) $$\begin{array}{cc} \; & E_{O}=\left[\begin{array}{cccccc} e_{11} & e_{12} & e_{13} & 0 & 0 & 0\\ \; & e_{22} & e_{23} & 0 & 0 & 0\\ \; & \; & e_{33} & 0 & 0 & 0\\ \; & \; & & e_{12} & 0 & 0\\ \; & \; & & \; & e_{13} & 0\\ \mathrm{sym}. & \; & & \; & & e_{23} \end{array}\right],\\ & e_{11}=\frac{2EA}{a^{2}}\left(1+1.52325\cdot k+0.129225\cdot \sigma \right),\\ \; & e_{12}=\frac{EA}{a^{2}}\left(0.845615\cdot k-0.105243\cdot \sigma \right),\\ \; & e_{13}=\frac{EA}{a^{2}}\left(1.26604\cdot k-0.153207\cdot \sigma \right),\\ \; & e_{22}=\frac{2EA}{a^{2}}\left(1+1.35912\cdot k+0.137028\cdot \sigma \right),\\ \; & e_{23}=\frac{EA}{a^{2}}\left(1.51283\cdot k-0.168813\cdot \sigma \right),\\ \; & e_{33}=\frac{2EA}{a^{2}}\left(1+0.921194\cdot k+0.16101\cdot \sigma \right). \end{array}$$

**Figure 11:**
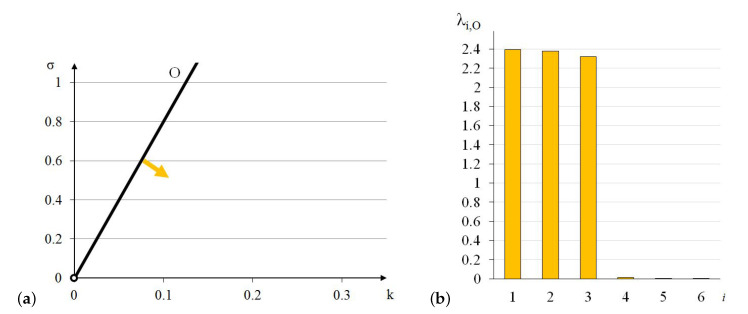
Extreme properties of the expanded octahedron: (**a**) Line of extreme properties σ=8.03·k. (**b**) Distribution of eigenvalues for k=0.1,σ=0.803 (multiplier $EA/a^{2}$).

In this case, the elastic matrix indicates that the expanded octahedron is an orthotropic module. Extreme properties of the module are presented in Figure 11.

The module can be identified as quasi trimode since one eigenvalue is close to zero and two others are much smaller than the other three. Three positive eigenvalues have similar values.

Analysis of the graphs (Figure 11) leads to the conclusion that the material based on the expanded octahedron modules, arranged in accordance with their infinitesimal mechanisms, would have three stiff and three soft (one purely soft with the corresponding eigenvalue less than 0.01% of $\lambda_{\max,O}$ and two quasi-soft with eigenvalues less than 0.7% of $\lambda_{\max,O}$) modes of deformation. Moreover, realization of such a material is possible with the currently available materials.

**Figure 12:**
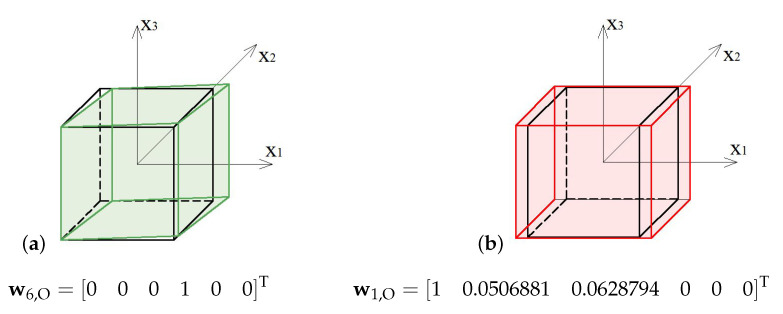
Deformation modes of the expanded octahedron: (**a**) Soft mode. (**b**) Stiff mode.

The soft mode (Figure 12a) of the expanded octahedron (O), represented by the eigenvector $w_{6,O}$, is a shear deformation in $x_{1}-x_{2}$ plane. The stiff mode (Figure 12b), represented by the eigenvector $w_{1,O}$, is volumetric with the dominant extension in $x_{1}$ direction.

### 4.4. Truncated Tetrahedron

**Figure 13:**
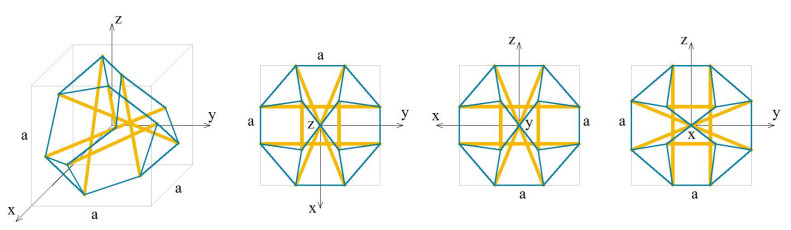
Geometry of the truncated tetrahedron module.

The analysed truncated tetrahedron module (T) is presented in Figure 13.

The elastic matrix obtained from the continuum model has a form:

(6) $$\begin{array}{cc} \; & E_{T}=\left[\begin{array}{cccccc} e_{11} & e_{12} & e_{12} & 0 & 0 & 0\\ \; & e_{11} & e_{12} & 0 & 0 & 0\\ \; & \; & e_{11} & 0 & 0 & 0\\ \; & \; & & e_{12} & 0 & 0\\ \; & \; & & \; & e_{12} & 0\\ \mathrm{sym}. & \; & & \; & & e_{12} \end{array}\right],\\ & e_{11}=\frac{2EA}{a^{2}}\left(0.836131+0.728014\cdot k+0.0709611\cdot \sigma \right),\\ \; & e_{12}=\frac{EA}{a^{2}}\left(0.232405+0.696312\cdot k-0.0709611\cdot \sigma \right). \end{array}$$

**Figure 14:**
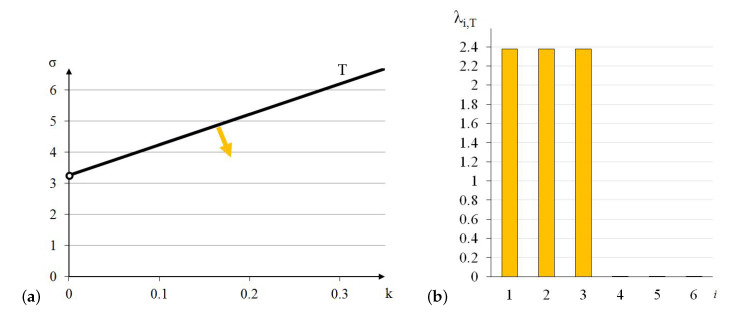
Extreme properties of the truncated tetrahedron: (**a**) Line of extreme properties σ=3.275+9.81·k. (**b**) Distribution of eigenvalues for k=0.1,σ=4.256 (multiplier $EA/a^{2}$).

Similarly to the previous module, the truncated tetrahedron is orthotropic. Extreme properties of the module are presented in Figure 14.

The module is trimode. Three positive eigenvalues are equal. Unfortunately, the values of σ are unreal as far as the currently available technology is concerned.

Analysis of the graphs (Figure 14) leads to the conclusion that the material based on the truncated tetrahedron modules, arranged in accordance with their infinitesimal mechanisms, would have three stiff and three purely soft (corresponding eigenvalues are less than 0.01% of $\lambda_{\max,T}$) modes of deformation. It is the most extreme material from all analysed examples. However, realization of such a material is not possible with the currently available materials.

**Figure 15:**
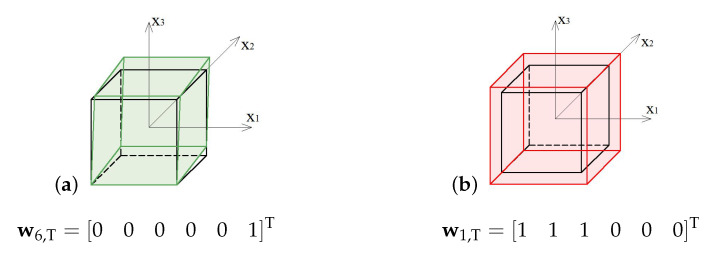
Deformation modes of the truncated tetrahedron: (**a**) Soft mode. (**b**) Stiff mode.

The soft mode (Figure 15a) of the truncated tetrahedron (T), represented by the eigenvector $w_{6,T}$, is a shear deformation in $x_{2}-x_{3}$ plane. The stiff mode (Figure 15b), represented by the eigenvector $w_{1,T}$, is volumetric with the uniform extension in all directions.

### 4.5. X-Module

**Figure 16:**
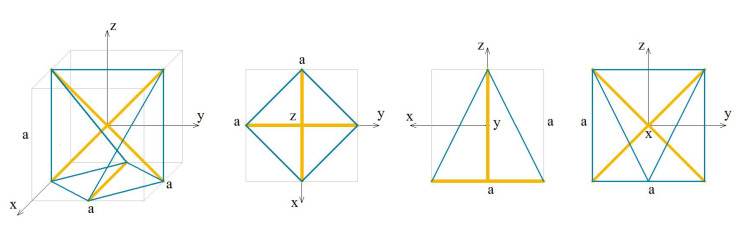
Geometry of the X-module.

The analysed X-module (X) is presented in Figure 16.

The elastic matrix obtained from the continuum model has a form:

(7) $$\begin{array}{cc} \; & E_{X}=\left[\begin{array}{cccccc} e_{11} & e_{12} & e_{13} & 0 & e_{15} & 0\\ \; & e_{22} & e_{23} & 0 & -e_{23} & 0\\ \; & \; & e_{33} & 0 & e_{15} & 0\\ \; & \; & & e_{12} & 0 & -e_{23}\\ \; & \; & & \; & e_{13} & 0\\ \mathrm{sym}. & \; & & \; & & e_{23} \end{array}\right],\\ & e_{11}=\frac{2EA}{a^{2}}\left(0.0441942+0.110947\cdot k-0.00988212\cdot \sigma \right),\\ \; & e_{12}=\frac{EA}{a^{2}}\left(0.0883883+0.0340207\cdot k+0.0395285\cdot \sigma \right),\\ \; & e_{13}=\frac{EA}{a^{2}}\left(0.0968935\cdot k-0.0197642\cdot \sigma \right),\\ \; & e_{15}=\frac{EA}{a^{2}}\left(-0.00850517\cdot k\right),\\ \; & e_{22}=\frac{2EA}{a^{2}}\left(0.0441942+0.193041\cdot k-0.0197642\cdot \sigma \right),\\ \; & e_{23}=\frac{EA}{a^{2}}\left(0.0340207\cdot k\right),\\ \; & e_{33}=\frac{2EA}{a^{2}}\left(0.0625+0.0484468\cdot k+0.00988212\cdot \sigma \right). \end{array}$$

**Figure 17:**
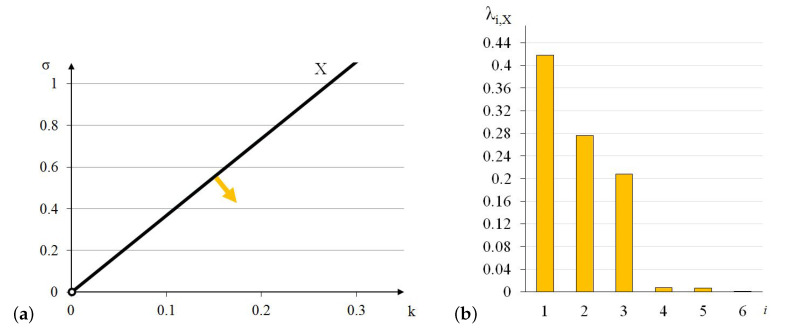
Extreme properties of the X-module: (**a**) Line of extreme properties σ=0.001+3.69·k. (**b**) Distribution of eigenvalues for k=0.1,σ=0.370 (multiplier $EA/a^{2}$).

The elastic matrix indicates that the X-module is anisotropic. Extreme properties of the module are presented in Figure 17.

The module can be identified as unimode since one eigenvalue is close to zero, despite two eigenvalues smaller than the other three. There is no dominant positive eigenvalue.

Analysis of the graphs (Figure 17) leads to the conclusion that the material based on the X-modules, arranged in accordance with their infinitesimal mechanisms, would have one stiff, one purely soft (corresponding eigenvalue is less than 0.01% of $\lambda_{\max,X}$) and four medium (two stiffer with the eigenvalues around 49% and 66% of $\lambda_{\max,X}$ and two softer with around 1.6% of $\lambda_{\max,X}$) modes of deformation. Moreover, realization of such a material is possible with the currently available materials.

**Figure 18:**
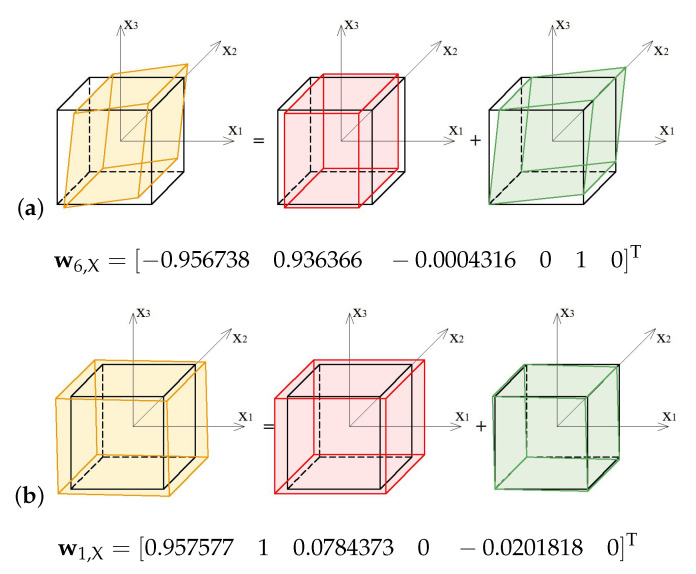
Deformation modes of the X-module: (**a**) Soft mode. (**b**) Stiff mode.

The soft mode (Figure 18a) of the X-module (X), represented by the eigenvector $w_{6,X}$, is a combination of a shear deformation in $x_{1}-x_{3}$ plane and a volumetric deformation with the dominant extension in $x_{1}$ and $x_{2}$ directions. The stiff mode (Figure 18b), represented by the eigenvector $w_{1,X}$, is a combination of a volumetric deformation with the dominant extension in $x_{1}$ and $x_{2}$ directions supplemented with a small extension in $x_{3}$ direction and a small shear deformation in $x_{1}-x_{3}$ plane.

### 4.6. Comparison of the Modules

**Figure 19:**
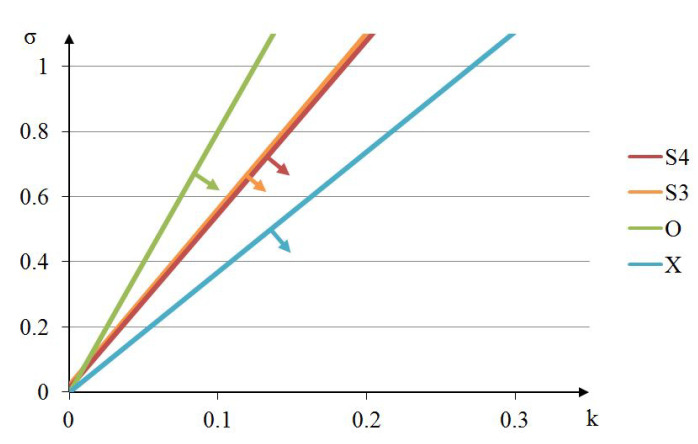
Lines of extreme properties for selected tensegrity modules.

Figure 19 depicts lines of extreme mechanical properties obtained for four out of five analysed tensegrity modules. As explained earlier, the line determined for the truncated tetrahedron goes beyond the range of achievable values of the parameter σ.

The biggest area where the elastic matrix E is positive definite is observed for the expanded octahedron and the smallest—for the X-module. The areas for both simplex modules are similar. From the point of view of commonly produced materials, the most real values of the parameters *k* and σ can be obtained for the X-module.

The eigenvalues for stiff modes that accompany the identified soft modes (Figure 5b, Figure 8b, Figure 11b, Figure 14b and Figure 17b) are on the same level for four modules: 4-strut simplex, 3-strut simplex, expanded octahedron and truncated tetrahedron, and much smaller for the X-module.

Soft deformation modes are volumetric with various signs for the 4-strut simplex, 3-strut simplex and expanded octahedron modules, but shear dominated for the truncated tetrahedron and the X-module. Stiff modes of deformation are more or less uniform volumetric for the 4-strut simplex, 3-strut simplex and truncated tetrahedron modules, volumetric in two directions for the X-module and volumetric in one direction for the expanded octahedron.

## 5. Conclusions

The main focus of the present paper is put on the identification of extreme mechanical properties of five regular tensegrity modules. The considered structures are assumed to be used as unit cells in 3D lattice metamaterials. The following tensegrity modules are analysed: 4-strut simplex, 3-strut simplex, expanded octahedron, truncated tetrahedron and X-module. It is shown that the material, in which all modules are arranged in accordance with their infinitesimal mechanisms, has the same mechanical properties as its unit cell and therefore, single tensegrity modules can be examined instead of more complicated multi-module systems.

Extreme features of the proposed metamaterials are identified with the use of the continuum model, which enables the authors to find the equivalent elasticity matrices of the examined structures. Analysis of these matrices leads to the determination of eigenvalues and corresponding eigenvectors which, on the other hand, enable the identification of soft and stiff deformation modes.

For each analysed module and thus for the metamaterial based on this module, a line of extreme mechanical properties is determined. It is a line on the plane k,σ, on which the smallest eigenvalue of the elasticity matrix is close to zero and which defines the possibility of the soft mode of deformation. Additionally, soft and stiff deformation modes are presented analytically and graphically for each unit cell.

The analyses indicate that both simplex modules are similar from the point of view of their extreme properties. They have one stiff, one purely soft, some quasi-soft and some medium deformation modes. The area where the elastic matrix is positive definite is almost the same for both modules. The 3-strut simplex has less struts and cables, but on the other hand, the 4-strut module can be used to create more regular rectangular patterns. The truncated tetrahedron module shows the most extreme properties, as it has three purely stiff and three purely soft deformation modes. However, it could not be created with the currently available materials. The expanded octahedron has three stiff, one purely soft and two quasi-soft modes of deformation and moreover, the biggest area of positive definiteness of the elastic matrix. The X-module has one stiff and one soft mode and the smallest area for which the elastic matrix is positive definite, but in the same time, it is quite simple to create, it has small amount of elements and may be formed in very regular patterns.

The obtained results are promising from the point of view of future creation of tensegrity lattices and metamaterials with extreme mechanical properties. A metamaterial based on the 4-strut simplex module is identified as quasi bimode, 3-strut simplex and expanded octahedron as a quasi trimode, truncated tetrahedron as a trimode and X-module as a unimode. However, the truncated tetrahedron module cannot be considered as a potential unit cell of the metamaterial, because the parameters *k* and σ, for which the soft mode is obtained are not achievable with the current technology. From the practical point of view, taking into account the available technology, a metamaterial based on the X-module has the greatest potential.
